# Case Report: Refractory Chronic Spontaneous Urticaria Treated With Omalizumab in an Adolescent With Crohn’s Disease 

**DOI:** 10.3389/fimmu.2021.635069

**Published:** 2021-03-02

**Authors:** Simona Barni, Mattia Giovannini, Giulia Liccioli, Lucrezia Sarti, Anna Gissi, Paolo Lionetti, Francesca Mori

**Affiliations:** ^1^Allergy Unit, Department of Pediatrics, Meyer Children’s University Hospital, Florence, Italy; ^2^Gastroenterology and Nutrition Unit, Meyer Children’s University Hospital, Florence, Italy

**Keywords:** anti-IgE monoclonal antibody, chronic spontaneous urticaria, inflammatory bowel diseases, Crohn’s disease, omalizumab, adolescent

## Abstract

Chronic spontaneous urticaria (CSU) is a mast cell-driven disease that is often associated with autoimmune or autoinflammatory conditions. Omalizumab is recommended in the treatment of refractory CSU in patients over 12 years of age who do not respond to four standard doses of antihistamines. Omalizumab blocks the mast cells’ degranulation, thus interrupting the resulting inflammatory cascade driven by T-helper 2 (Th2) cytokines. The efficacy of omalizumab in controlling CSU and possible associated diseases has been studied in few patients so far. In particular, some case reports describe adults with CSU and concomitant inflammatory bowel diseases (IBD), such as Crohn’s disease (CD) or ulcerative colitis (UC). Although the treatment of CD with anti-tumor necrosis factors-α (TNF-α) seems to be effective in controlling CSU, no cases of the utility of omalizumab in patients with both conditions have been described so far. At the moment, there is no evidence that the pathogenetic mechanisms underlying CD are linked to the same pathways that are inhibited by omalizumab for the treatment of CSU. We present the first pediatric case of refractory CSU and CD in which omalizumab led to CSU remission, even if the follow-up period was limited. In conclusion, our experience shows how CSU could be associated with CD and successfully treated with the monoclonal anti-IgE antibody in a patient on immunosuppressive therapy. However, more data is needed from a larger population.

## Introduction

Omalizumab is a recombinant monoclonal antibody (mAb)—direct against the Fcϵ portion of the immunoglobulin (Ig)E antibodies—that blocks interaction with the high-affinity receptors (FcϵRI) expressed on the surface of target cells such as basophils and mast cells and that, consequently, blocks their release of several mediators (1).

Omalizumab acts mainly on a T-helper 2 (Th2) inflammation with a prominent role on mastocytes by inhibiting their degranulation and interrupting the resulting inflammatory cascade driven by Th2 cytokines (2). The efficacy of omalizumab has been proven in several diseases with a high level of evidence (i.e., allergic asthma, chronic urticaria, allergic rhinitis), medium level of evidence (i.e., nasal polyposis, facilitation of oral food allergen immunotherapy, facilitation of subcutaneous immunotherapy to aeroallergens, non-allergic asthma, allergic bronchopulmonary aspergillosis), and low level of evidence (i.e., mast cell activation syndrome, idiopathic anaphylaxis, atopic dermatitis, eosinophilic esophagitis, aspirin-exacerbated respiratory disease, asthma-chronic obstructive pulmonary disease overlap) (3).

In particular, the utility and safety of omalizumab in the treatment of severe allergic asthma has been known for many years. Indeed, it was approved by the United States Food and Drug Administration (FDA) in 2003, and 2 years later by the European Medicine Agency (EMA).

Moreover, the European Academy of Allergy and Clinical Immunology (EAACI), European Dermatology Forum (EDF), Global Allergy and Asthma European Network (GA2LEN), and World Allergy Organization (WAO) recommended the use of omalizumab in step three of the treatment for chronic spontaneous urticaria (CSU) in patients 12 years or older (3). CSU is defined by the daily appearance of wheals and/or angioedema, without an identified trigger, for a period lasting at least 6 weeks (3).

Autoimmunity seems to play a role of paramount importance in CSU, which is frequently associated with other autoimmune diseases, especially thyroiditis and celiac disease (4). Recently, few reports describe CSU associated with autoinflammatory gastrointestinal diseases, such as ulcerative colitis (UC) and Crohn**’**s disease (CD), especially in adult patients (5–8).

We describe the first pediatric case of a girl with CD who developed refractory CSU and required treatment with omalizumab, leading to CSU remission.

## Case Report

**Figure 1:**
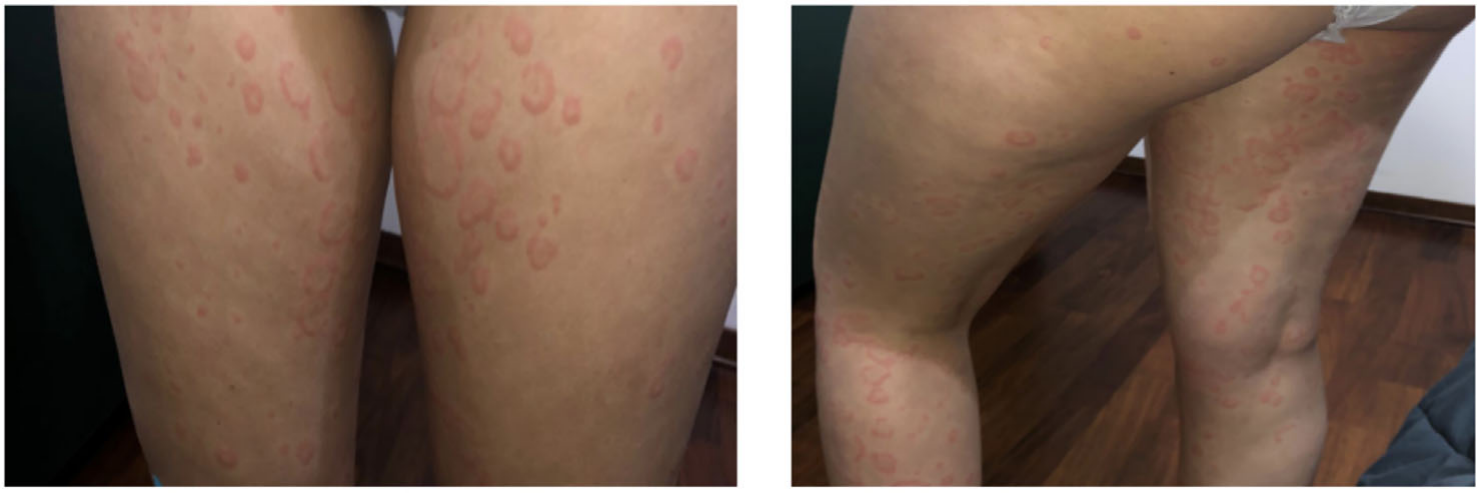
Urticaria on the thighs and legs of the patient.

We present the case of a Caucasian girl who has been suffering from CD since she was 12 years old. The colonoscopy revealed linear millimetric ulcerations on slightly hyperemic mucosa in the terminal ileum; the ileocecal valve presented rounded ulceration on the proximal edge; the mucosa of the whole colon up to the rectum was normal in appearance. The histological exam showed that, at the level of the terminal ileum and ileo-cecal valve, architecture of villi was within normal limits without an increase in intraepithelial T lymphocytes with pseudo-atrophic aspects and erosions of the epithelial lining. In the lamina propria, an increase in the inflammatory component, partly in the form of hyperplastic follicular lymphoid aggregates, partly in the neutrophilic and eosinophilic component, involved in some points the glandular structures without creating cryptic abscesses. There were no giant cells or granulomas. The cecum, the ascending-transverse-descending colon, the sigma and rectum were of normal morphology. At 12 years old, she started therapy with azathioprine and mesalazine without any disease relapse. At 17 years of age, she began to present episodes of angioedema, which were, in the beginning, isolated and, after 6 months, associated with urticaria. The patient had not undergone any treatment change for CD in the previous 5 years. In January 2019, she was referred to our Allergy Unit as urticarial episodes occurred daily (**Figure 1**) and persistently for more than 6 weeks despite being treated with second-generation non-sedating H1-antihistamines (sgAH1) up to 2 fold the approved doses. On the physical examination, she presented diffuse wheals, especially on the trunk and legs but sparing the face, in the absence of angioedema or other relevant clinical manifestations, including articular or musculoskeletal ones. Moreover, no clinical features of urticarial vasculitis were detected, which allowed us to rule it out. A full diagnostic work-up for chronic urticaria was performed (**Table 1**). She underwent a gastroenterology visit with esophagogastroduodenoscopy and colonoscopy with retrograde ileoscopy, which ruled out a relapse of CD. Moreover, the fecal calprotectin and the erythrocyte sedimentation rate (ESR) were in the normal range (**Table 1**). Therefore, she was diagnosed with CSU and angioedema in a patient with CD in remission.

**Table 1:** Laboratory workup.

Parameters, unit of measure	January 2019	February 2020	Normal range
CBC:			
- WBC, n/mm^3^ - EOS, n/mm^3^	5,310 63.7	4,980 81.2	4,000–10,000 30–350
AST, U/L	14	16	1–31
ALT, U/L	10	9	1–31
Creatinine, mg/dl	0.65	0.60	0.50–1.20
CRP, mg/dl	0.6	0.5	0–0.7
ESR, mm/hr	13	15	2–37
Specific IgE to common airway (mites, molds, animal epithelia and grass, weed, birch, olive, cypress pollens) and common food allergens (milk, egg, wheat, cod, tomato, soy, peanut), KU/l	<0.10	np	<0.10
HP-Abs U/ml	0.10	np	<0.30
TSH μlU/ml	3.13	3.01	0.35–4.94
fT4, pg/ml	8.8	8.5	6-12
TG-Ab, IU/ml	1	1	<4
TPO-Ab, IU/ml	1	1	<9
tTG-IgA, U/ml	0.4	1	<7
ANA	neg	neg	<1:80
C3, mg/dl	80	np	66–185
C4, mg/dl	35	np	15-52
C1-INH quantitative, g/l	0.22	np	0.15–0.33
C1-INH functional, %	100	np	70–100
Fecal calprotectin, μg/g	30	20	<70

ANA, antinuclear antibodies; ALT, alanine aminotransferase; AST, aspartate aminotransferase; C-reactive protein; CBC, complete blood count; C1-INH, C1 esterase inhibitor; CRP, C-reactive protein; EOS, eosinophils; ESR, erythrocytes sedimentation rate; fT4, free-thyroxine; HP-Abs, Helicobacter pylori-antibodies; IgE, Immunoglobulin E; neg, negative; np, not performed; TG-Ab, thyroglobulin-antibodies; TPO-antibodies, thyroid peroxidase-antibodies; TSH, thyroid-stimulating hormone; tTG-IgA, transglutaminase-Immunoglobulin A-antibodies; WBC, white blood cell.

**Figure 2:**
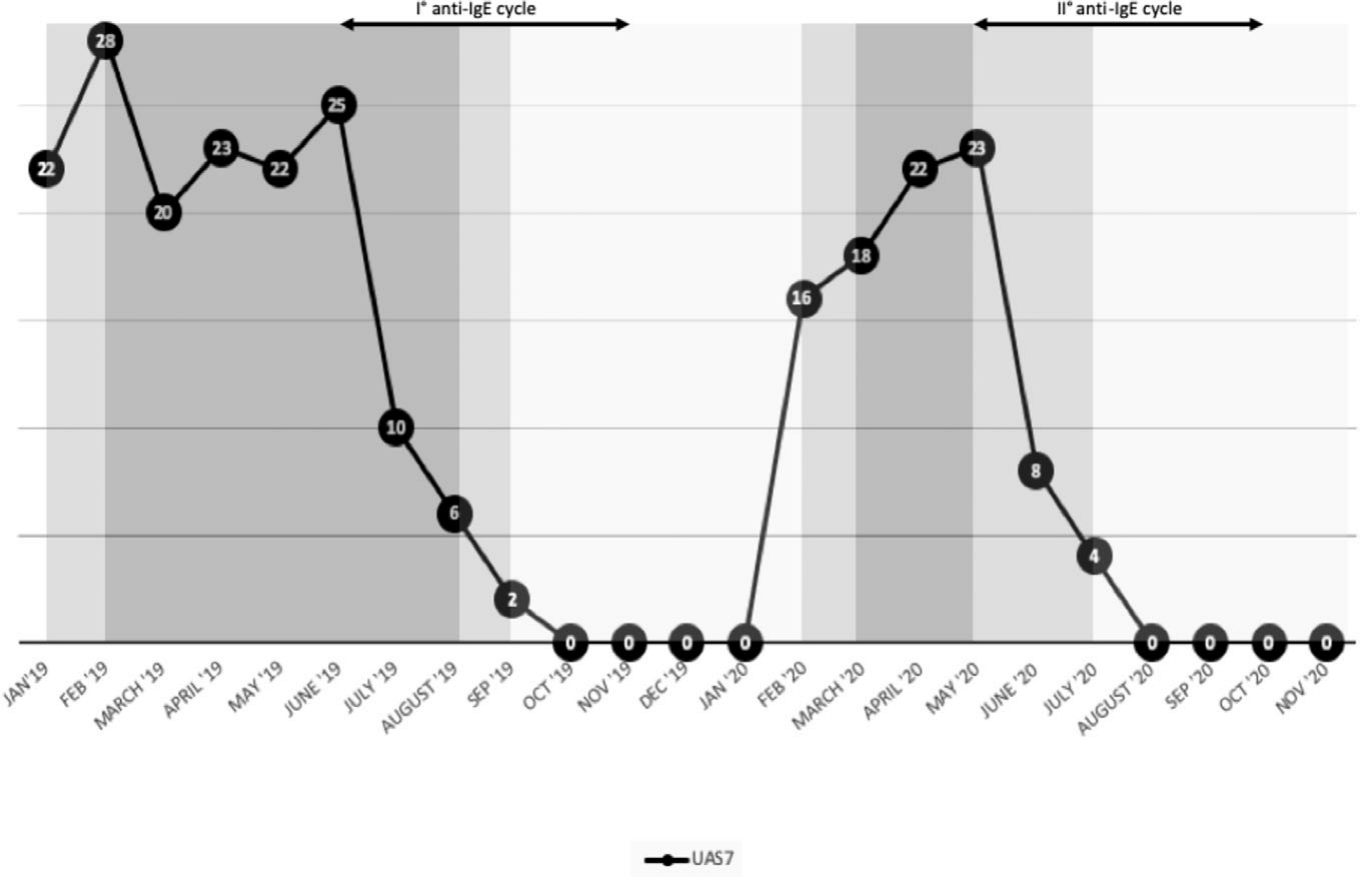
Effect of second-generation not sedating H1-antihistamines and omalizumab on the clinical manifestation of chronic spontaneous urticaria described through 7-day Urticaria Activity Score (UAS7).

We suggested maintaining the dose of sgAH1 twice a day and monitoring the disease activity by filling out the seven-day Urticaria Activity Score weekly (UAS7) as recommended by the EAACI/GA2LEN/EDF/WAO guideline (9). According to our center**’**s clinical guidance and as recently explained in a published article by Sarti et al., we adjusted the dose of the sgAH1 therapy based on the patient**’**s UAS7. Specifically, if the UAS7 was higher than 15 for at least 2 weeks, a step-up in therapy was performed. If it was 1–15, the sgAH1 dose was maintained. Finally, if it was 0 for at least 2 weeks, a step-down in therapy was performed (10). After 4 weeks, the patient was evaluated again through telemedicine; due to the low disease control, we suggested increasing the sgAH1 dose to three times a day (**Figure 2**). Taking into account the low control of urticaria despite the sgAH1 treatment at threefold, the approved doses and the occurrence of somnolence as a side effect of antihistamines at high doses, in June 2019, we started treatment with subcutaneous omalizumab (300 mg) every 4 weeks (**Figure 1**). The patient continued taking sgAH1 three times a day for the first two months of treatment with the monoclonal antibody. As her UAS7 progressively improved, the sgAH1 dose was tapered to two times a day and to once a day in September 2019 (**Figure 2**). After the sixth injection (November 2019), omalizumab was stopped for two months according to the therapeutic schedule approved in Italy. The patient continued to take only one dose of cetirizine until February 2020, when a relapse of urticaria**’**s and angioedema**’**s clinical manifestations occurred. For this reason, she was given a twofold increase in the standard dosage of her sgAH1 treatment. Also, to exclude a reactivation of CD, she underwent new tests (**Table 1**), which gave normal results. Because of the poor management of urticaria, sgAH1 was prescribed three times a day for a short period of time. Because of the poor tolerance to the latter therapy, in May 2020, we started the second cycle of treatment with subcutaneous omalizumab (300 mg) every 4 weeks (**Figure 2**). Through telemedicine, we decided to progressively reduce sgAH1 to twice a day and finally once a day from September 2020. In October 2020, she underwent the last omalizumab injection. From August to October 2020, the registered UAS7 was 0 (**Figure 2**). After one month from the last omalizumab injection, the urticaria and angioedema were under control with sgAH1 once a day, and UAS7 was still 0. Moreover, the patient received 12 injections of omalizumab without reporting any side effects.

## Discussion

In this case report, we highlighted the efficacy and safety of omalizumab in an adolescent with refractory CSU and CD in remission on immunosuppressive therapy. The use of monoclonal anti-IgE therapy in the immunosuppressed host is limited to a few case reports, including adult patients with the hyper-IgE syndrome (HIES) (11) and HIV infection (12) and an adolescent with a common variable immunodeficiency (CVI) (13). In particular, Bard et al. (11) reported the first case of a 26-year-old woman with HIES with severe recalcitrant eczematous dermatitis, which was successfully treated with high-dose monoclonal anti-IgE therapy. Moreover, Iemoli et al. (12) described a case of excellent tolerability and efficacy of omalizumab in the treatment of CSU in a 56-year-old HIV-positive man on antiretroviral therapy; in particular, the viral load remained undetectable, and the CD4^+^ T cell counts improved. Recently, Comberiati et al. (13) reported the first case of a 19-year-old female with CVID treated successfully in terms of efficacy and safety with omalizumab for CSU after a non-effective trial with intravenous immunoglobulin at immunomodulatory dosage. The remission of cutaneous symptoms was obtained after the first omalizumab injection and persisted after the 12-month follow-up period.

As already described, CSU is a mast cell-driven disease (14). Two groups of mast cell degranulating signals have been described so far: IgE auto-antibodies to auto-allergens (type I autoimmunity) and IgG or IgM autoantibodies targeting activating mast-cell receptors (type II autoimmunity) (15). These two types of autoimmune mechanisms of skin mast cell degranulation are considered to be relevant causes of CSU in most patients (15). Indeed, CSU has been associated with numerous autoimmune diseases, including inflammatory bowel diseases (IBDs) (4). However, very few case reports of CSU or angioedema in adult patients associated with IBDs have been described so far (5–8).

Farkas et al. and Freeman (5, 6) described two cases of hereditary angioedema (HAE) associated with CD: two males aged 35 and 29. The particularity of the latter clinical case (6) was that the patient**’**s mother suffered from the same diseases—HAE and CD—that are, apparently, two unrelated conditions. Nevertheless, in this case, they appeared to be genetically linked. Mansueto et al. (16) reported the case of a 64-year-old man with CSU who presented signs and symptoms of subclinical CD, a manifestation that had never been previously described in these patients.

Currently, the action mechanism of omalizumab is not fully understood. It is known that it binds to free IgE, thus lowering free IgE levels and their receptors (17). In the literature, the use of omalizumab to treat CSU in patients with concomitant IBDs is limited to a few case reports. In particular, Witten et al. (18) described the successful use of omalizumab in a 23-year-old male with triple immune/autoinflammatory disease: CSU and angioedema associated with CD and familial Mediterranean fever (FMF). Grieco et al. (19) reported the case of a 49-year-old woman with UC who was under mesalazine treatment. She had autoimmune thyroiditis, chronic hypereosinophilia and CSU plus Besnier**’**s prurigo treated with omalizumab with the resolution of urticaria and improvement of cutaneous clinical manifestations of prurigo.

The CSU and CD have a strong autoimmune involvement with an increase of proinflammatory cytokines (20). One common thread in the pathophysiology of both diseases is the imbalance in cytokine levels, in particular for IL-17 and TNF-α (21–23). Habal et al. (24) described the first case of CSU with angioedema coexistent with CD that was successfully treated with anti-TNF-α agents. Whether the CSU was cured due to the remission of the CD or because the TNF- α was a common cytokine in the pathogenetic pathway of the two diseases is difficult to understand. The authors hypothesized that given the similarity of cytokine derangements in CSU and CD, therapies that target the TNF- α could be effective in both conditions (24). In support of this hypothesis, cases of patients with refractory CSU, who were successfully treated with TNF-α inhibitors, are described in the literature (25, 26). Conversely, so far, no studies have concluded that anti-IgE therapy could improve both CSU and CD. Indeed, although CSU and CD are autoimmune diseases sharing the imbalance of some cytokines, when present in the same patient, they appear to be two concomitant diseases, and one is not the cause of the other.

In conclusion, even if the follow-up period of our case was limited and more data would be needed on more extensive populations, our experience shows how CSU could be associated with CD and successfully treated with monoclonal anti-IgE antibody even in a patient on immunosuppressive therapy.

## Data Availability Statement

The original contributions presented in the study are included in the article/supplementary material. Further inquiries can be directed to the corresponding author.

## Ethics Statement

Written informed consent was obtained from the minor(s)**’** legal guardian/next of kin for the publication of any potentially identifiable images or data included in this article.

## Author Contributions

SB, MG, GL, and LS conceptualized the work. SB, MG, FM, AG, and PL drafted the manuscript. SB and FM performed the investigations and critically revised the manuscript. All authors contributed to the article and approved the submitted version.

## Funding

The research did not receive any specific grant from funding agencies in the public, commercial, or not-for-profit sectors. The publication fee was financed by Novartis. The funder was not involved in the study design, collection, analysis, interpretation of data, the writing of this article or the decision to submit it for publication.

## Conflict of Interest

The authors declare that the research was conducted in the absence of any commercial or financial relationships that could be construed as a potential conflict of interest.
